# Prevalence of self-medication with antibiotics and associated factors in the community of Asmara, Eritrea: a descriptive cross sectional survey

**DOI:** 10.1186/s12889-019-7020-x

**Published:** 2019-06-10

**Authors:** Yonatan Ateshim, Batseba Bereket, Feruz Major, Youel Emun, Biruck Woldai, Ismail Pasha, Eyasu Habte, Mulugeta Russom

**Affiliations:** 1 School of Pharmacy, Asmara College of Health Sciences, Asmara, Eritrea; 2 Biostatistics and Epidemiology Unit, School of Public Health, Asmara College of Health Sciences, Asmara, Eritrea; 3 Eritrean Pharmacovigilance Centre, National Medicine and Food Administration, Ministry of Health, Asmara, Eritrea

**Keywords:** Self-medication, Antibiotics, Prevalence, Asmara, Community

## Abstract

**Background:**

Development of drug resistance caused by self-medication with antibiotics, can be seen as one of the growing global threats. Self-medication is defined as the selection and use of medicines by individuals to treat self-recognized illnesses or symptoms. The purpose of this study is to assess the practice of self-medication with antibiotics and associated factors among the community of Asmara, Eritrea.

**Methods:**

This was a community based descriptive cross-sectional study conducted in 16 selected sub-districts of Asmara from September to November 2017. A Two-stage cluster sampling was employed to select study sites and participants. Data was collected in a face to face interview with a structured questionnaire and entered to CSPro version 6.2. Descriptive statistics, cross-tabulation and logistic regression were executed using SPSS version 22.

**Results:**

A total of 580 study participants were recruited with a response rate of 99.5% (*N* = 577). The prevalence of Self-medication with antibiotics (SMA) in this study was found to be 45.1% [95% CI (40.5, 49.6)] and majority of them practiced once or twice in a period of 12 months. The main reasons for SMA were previous successful experience (34.4%) and the illness being ‘not serious enough to seek medical care’ (25.7%). Of those who self-medicated, 84.1% of used amoxicillin at least once. Wound infection (17.9%) and sore throat (13.9%) were the most self-recognized complaints that required self-medication. Antibiotics were supplied and recommended mostly by the community drug outlets. Only Sex (*p =* 0.046), knowledge (*p =* 0.019) and attitude (*p < 0.001)* of the participants were found significantly associated with the practice of SMA in the multivariate logistic regression.

**Conclusions:**

Though majority of the respondents considered self-medication with antibiotics as inappropriate practice, about half of them were practicing it anyway. Therefore immediate attention from relevant bodies is required.

**Electronic supplementary material:**

The online version of this article (10.1186/s12889-019-7020-x) contains supplementary material, which is available to authorized users.

## Background

Self-medication with antimicrobial is frequently noted as one of the major factors contributing to drug resistance [1]. The World Health Organization (WHO) defines self-medication as “the selection and use of medicines by individuals to treat self-recognized illnesses or symptoms” [2]. Antibiotics are substances produced by a microorganism, or a chemical synthesis, which in low concentrations can inhibit the growth and/or kill bacteria [3]. Unlike other drugs and virtually all other technologies, antibiotics suffer from transmissible loss of efficacy over time [4].

Antibiotic resistance refers to the phenomena when an antibiotic, which at its therapeutic level was once able to effectively stop the growth of the bacteria, has lost its ability to do so [5]. Self-medication with and overuse/misuse of antibiotics have been identified among the main risk factors for antibiotic resistance [6]. Moreover; lack of knowledge is a major factor responsible for inappropriate antimicrobial use and hence resistance globally [6].

Antibiotic resistance may result in prolonged illnesses, more health facility visits, extended hospital stays, the need for more expensive medications, and even death [7]. If the current trend continues, 10 Million deaths are attributable to AMR worldwide by 2050 [8].

A retrospective study conducted in Eritrea in 2016, regarding bacterial pathogen resistance to antimicrobials, showed an overall growth of resistant bacteria is 37.4% [9]. One of the effective strategies to prevent AMR had been staying one step ahead of the pathogens through discovery of new antibiotics. This could no longer be as productive since 15 out of the 18 largest pharmaceutical companies, owing to the financial burden over other drugs such as those used for chronic illnesses, abandoned the antibiotic field [10]. The only weapon to save the currently effective antibiotics from developing resistance is therefore handling them with care.

This study was conducted to establish the status of SMA among the community of Asmara. Moreover, it is aimed at identifying common perceived illnesses that required SMA, determining commonly used antibiotics, finding out the sources of information as well as antibiotics for the practice of SM. And the results from this study are expected to help in the planning of educational and regulatory interventions to promote the rational use of antibiotics.

## Methods

### Study designs and setting

This was a descriptive cross-sectional study conducted in selected sub-districts of Asmara, Eritrea from September to November 2017. Asmara is the capital city of Eritrea with 13 districts and 37 sub-districts.

### Source and study population

The source population of this study were the residents of Asmara. According to the Municipality of Asmara, during the study period (2017) there was a total of 108, 896 households and a population of 422,309 distributed in an area of 44.99 km^2^.

Randomly selected residents of Asmara aged 18 years and above who were willing to participate in the study and without any hearing, speech or mental disability were included in the study population.

### Sample size determination

Sample size was calculated using the single proportion formula without correction for continuity n = Z^2^P (1-P)/d^2^. At 95% confidence interval, the Z statistic value is 1.96 and P, estimated value for the particular indicator, was determined to be 0.39 from a pilot study. Assuming degree of precision (in proportion of one, d = 0.05), and a 5% non-response rate and finally adjusting by considering design effect (1.5), the sample size (n) was found to be 577 persons.

### Sampling technique

A two-stage cluster sampling was employed and sixteen out of the total 37 sub-districts of Asmara were selected.

In the first stage, the sub-districts were the sampling units. Probability proportionate to size sampling technique that gives the highest share and hence, chance of selection to the sub-districts with the highest number of households they consist was used.

In the second stage, the participants (head of the households or any family member of it in the absence of the head) from the selected sub-districts were identified using systematic random sampling.

### Data collection tool and approach

A structured questionnaire (Additional file 1) was developed on the basis of similar study conducted previously [11]. The questionnaire consisted both close-ended and open-ended questions and encompassed two parts. The first part inquired about the sociodemographic characteristics, whereas the second part was designed to capture data about previous self-use of antibiotics in the past 12 months (during initiation of the study), condition(s) for which antibiotics were self-prescribed, source of antibiotics, source of information, name(s) of antibiotics used. Moreover, few questions were included to probe the knowledge about antibiotics and attitude toward self-medication with antibiotics (SMA).

Selected participants were interviewed for about 15-20 minutes in their respective residence. Antibiotics commonly used in Eritrea were used as samples to help participants recall the name of the antibiotics they took. Data collection was conducted during the weekends or in the evening of the weekdays to include male participants who would leave their homes for work, as observed from the pilot study.

### Data processing and statistical analysis

Data was entered into CSPro version 6.2 and was analyzed with SPSS version 22. First, descriptive analysis was performed using frequency, percentage, median and interquartile range. Cross-tabulation was then carried out to look for possible association between self-medication with antibiotics and the independent variables. Finally, univariate and multivariate logistic regression at 95% CI were computed. Variables found to be significant at univariate level were included in the multivariate logistic regression analysis to control other potential confounders. Statistical significance for all analyses was set at *p* < 0.05.

### Operational definitions

#### Knowledgeable and unknowledgeable

Participants whose answers to the questions about what antibiotics are, what they are used for, and whether antibiotics could treat common cold or not were correct and, continued till the completion of course of treatment with antibiotics were classified as Knowledgeable. Any incorrect answer for any of these questions and discontinuing before completion of the regimen renders the respondent Unknowledgeable.

#### Positive attitude and negative attitude

Respondents were asked on what they think of self-medication with antibiotics. Those who had a notion that self-medication with antibiotics is inappropriate practice were considered as having positive attitude. Whilst, those who had a belief that it is unacceptable practice were categorized as respondents with negative attitude.

## Results

### Socio-demographic characteristics and prevalence of self-medication with antibiotics

A total of 580 study participants were recruited and this yielded a response rate of 99.5% (*N* = 577). The study participants were dominated by females (58.8%) and the median age was37 years (IQR = 24) (Table 1). Majority (27.9%) of participants were in the age group of 25–34 years and 61.9% of them were married. A quarter of the study participants (24.3%) had secondary level of education and the median monthly income was 1500 (IQR = 1650).

**Table 1:** Socio-demographics of the participants

Variables	Frequency	Percentage	Prevalence %	CI at 95%
Gender				
Male	238	41.2	55	[47.9, 62.2]
Female	339	58.8	38.3	[32.5, 44.0]
Age				
< 24	111	19.2	60.4	[50.5, 70.4]
25–34	161	27.9	43.1	[34.0, 52.3]
35–44	125	21.7	12.2	[32.4, 51.9]
45–54	74	12.8	12.9	[30.3, 55.4]
> 55	106	18.4	36.0	[25.8, 46.1]
Median age	37			
IQR	24			
Marital status				
Single	163	61.9	59.3	[50.9, 67.7]
Married	357	28.2	38.8	[33.1, 44.5]
Divorced	23	4.0	47.4	[22.6, 72.1]
Widowed	34	5.9	38.7	[20.5, 56.9]
Educational level				
Illiterate	32	5.5	34.8	[13.7, 55.8]
Primary	64	11.1	34.0	[20.8, 47.1]
Junior	95	16.5	35.2	[23.8, 46.6]
Secondary	246	42.6	45.0	[38.1, 52.0]
College	140	24.3	58.1	[49.0, 67.2]
Occupation				
Governmental	170	29.5	46.1	[37.8, 54.4]
Private service	58	10.1	47.9	[33.3, 62.6]
Self-employed	58	10.1	47.7	[32.4, 63.1]
Unemployed	60	10.4	50.0	[35.0, 65.0]
House wife	191	33.1	35.8	[28.0, 43.5]
Student	40	6.9	66.7	[50.5, 82.8]
Monthly income				
Non incomers^a^	291	50.4	43.3	[36.9, 49.8]
≤ 1000	92	15.9	41.3	[29.9, 52.7]
1001–2500	136	23.6	48.2	[38.9, 57.6]
> 2500	58	10.1	52.3	[36.9, 67.6]
Median	1500			
IQR	1650			

Note: *CI* Confidence Interval, *IQR* Interquartile range

^a^ Unemployed, housewife, student

The prevalence of self-medication with antibiotics in the past 12 months prior to the data collection was found to be 45.1% [95% CI (40.5, 49.6)]. As depicted in Table 1, The Median of SMA practice was 1 (IQR=1), the maximum was 8 times and majority of the respondents practiced once or twice during the 12 months period.

### Perceived complaints and reasons for self-medication with antibiotics

**Fig. 1:**
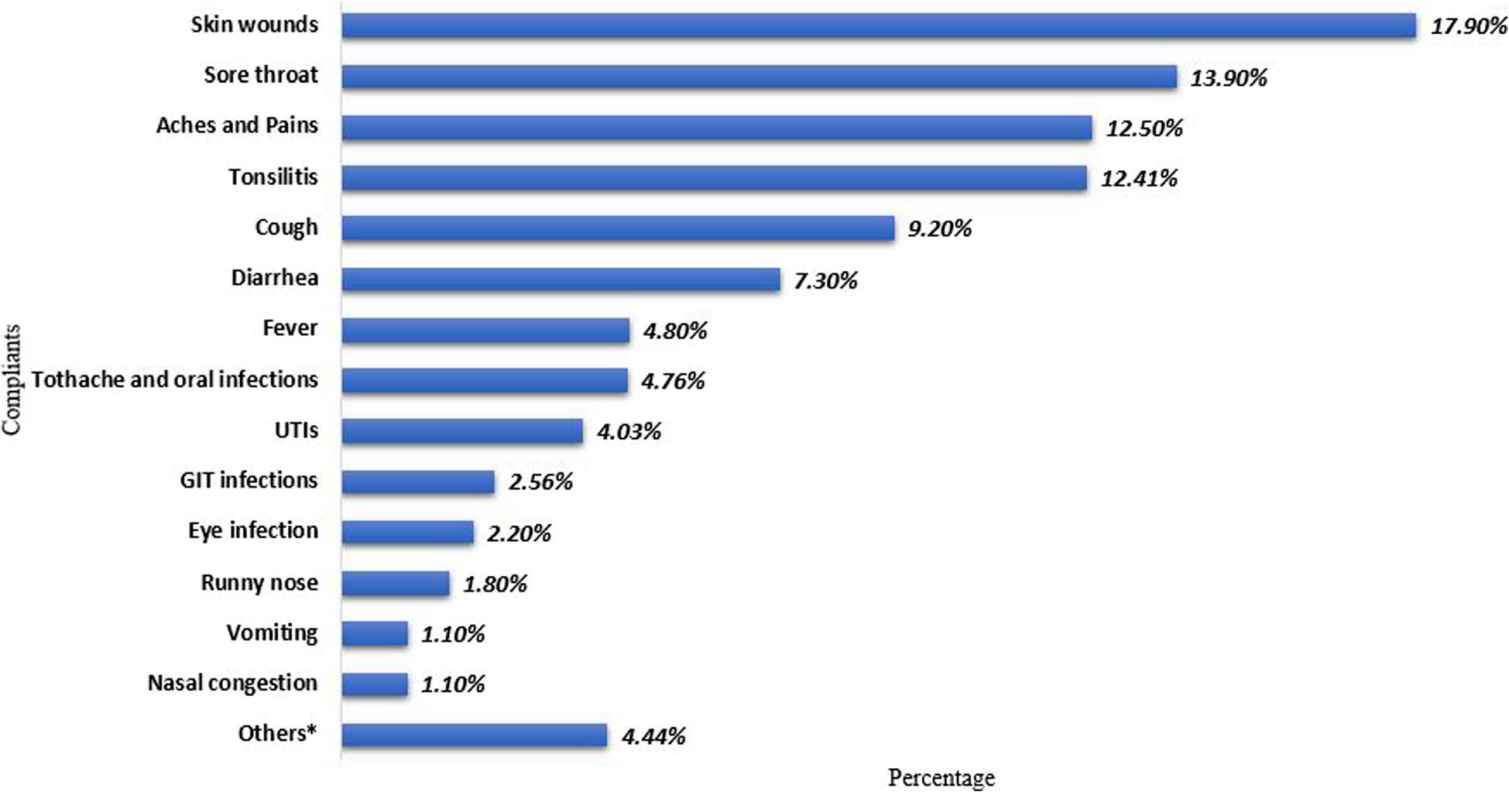
Perceived complaints that required SMA. Others include broken bone, dysentery, internal illness, knee injury, leg infection, post labor infection, RTIs, and whitlow

**Fig. 2:**
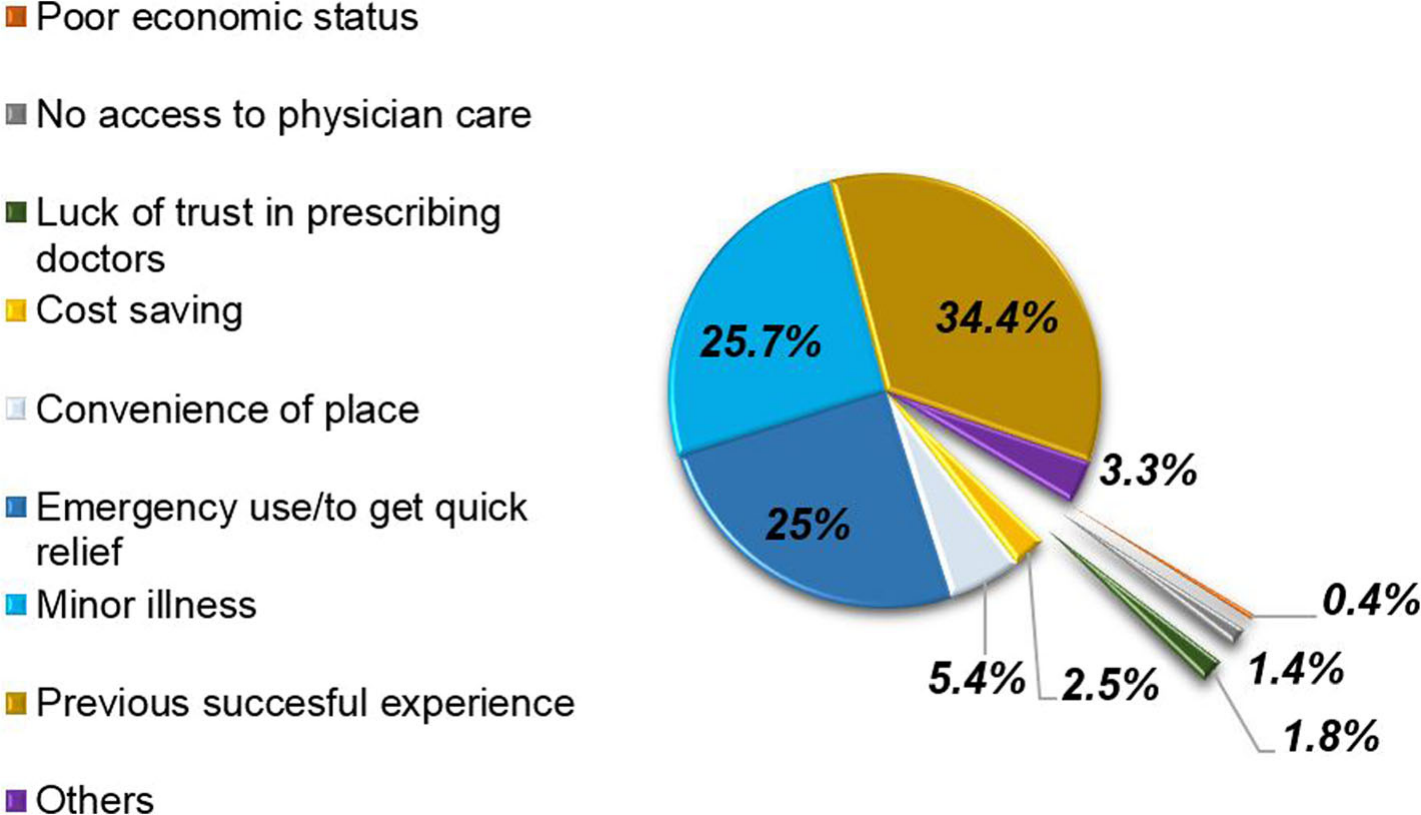
Reasons for self-medication with antibiotics. Others include easy availability of antibiotics, frequency of illness, practitioner being a health professional, to save time

Self-medication with antibiotics was practiced for wound infection (17.9%), sore throat (13.9%), aches and pains (12.5%), Tonsillitis (12.41%), cough (9.2%), diarrhea (7.3%), fever (4.8%) etc. (Fig. 1). The main reasons for self-medication with antibiotics were previous successful experience (34.4%), the illness being minor to seek medical attention (25.7%) and with intention of getting quick relief/ for emergency use (25%) (Fig. 2).

### Frequently used antibiotics and their source of antibiotics

Of those who self-medicated, 84.1% used amoxicillin at least once in the 12 months period. It was followed by ciprofloxacin (6.7%), tetracycline (2.51%), co-trimoxazole (2.09%), metronidazole (1.67%) etc. Antibiotics used for self-medication were obtained mainly from pharmacy retail outlets (68.0%), leftovers (15.2%), friends and/or relatives (10.4%) and sent from abroad (6.4%). Their source of information for the use of the antibiotics among others were pharmacy professionals (46.9%), previous doctor’s prescription (28.5%), friends/ relatives (8.8%), and internet/mobile applications (5.3%). Additionally, of those who self-medicated, 10.1% guessed the dosage of the antibiotics.

### Knowledge, attitude and practice of the respondents

Of all the study participants, 466 (80.8%) of them at least once in their life took antibiotics either prescribed by a qualified clinician or self-prescribed. Less than half (42.6%) of the participants completed their course of treatment as recommended. About a quarter (23.9%) of those who self-medicated disclosed that they discontinued their antibiotics when symptoms disappeared, 6.2% when they felt better and 2.3% stopped it after few days regardless of the outcome (Table 2).

**Table 2:** Course of treatment with antibiotics

When do you stop taking the antibiotics?	Frequency	%
After a few days regardless of the outcome	11	2.3
After the symptoms disappeared	115	23.9
A few days after the recovery	30	6.2
After antibiotics run out	110	22.9
At the completion of the course of treatment	205	42.6
After consulting a medical personnel/ pharmacist	9	1.9
For one day	1	0.2

Out of the 210 respondents who self-medicated, 18 (8.6%) respondents admitted they changed the dose of the antibiotics they used for self-medication every time they practice SMA, whereas 31(14.8%) quoted they sometimes deliberately changed the dose for the following reasons: condition improved (71.2%), condition worsened (11.5%), drug insufficient for complete treatment (7.7%), to reduce ADRs (7.7%) and 1.9% of them believed they were knowledgeable to decide to change the dose. Majority (76.7%) of them however, have never changed the dose of the antibiotics they used during the course of the practice of SMA.

About three-fourth (70.7%) of the study participants had a belief that use of self-medication with antibiotics is inappropriate (had positive attitude) while the rest (29.3%) believed that self-medication with antibiotics is acceptable practice (Table 3). Overall, 84.7% of the participants had inadequate knowledge on antibiotics and only 15.3% were found to be knowledgeable.

**Table 3:** The respondents’ responses regarding the knowledge and attitude questions

	Frequency	%
Do you know what antibiotics are?		
Yes	422	73.1
No	155	26.9
What do you think about SMA?		
Good practice	39	6.8
Acceptable practice	130	22.5
Not acceptable practice	408	70.7
Are antibiotics good for common cold?		
Yes	128	22.2
No	281	48.7
Don’t know	168	29.1
What are antibiotics used for?		
Bacterial infection	277	43.8
Viral infection	82	13.0
Bacterial and Viral infections	53	9.2
Don’t know	270	42.7
^a^Others	3	0.5

^a^ Fungal infections, helminthic infections, skin wounds

### Factors associated with self-medication practices

Being male (adjusted OR = 1.81; 95%CI: 1.01, 3.26), inadequate knowledge (adjusted OR = 2.13; 95%CI: 1.12, 4.05) and having negative attitude (OR = 7.47; 95%CI: 4.54, 12.29) were found to be significantly associated with self-medication of antibiotics (Table 4).

**Table 4:** The relationship between SMA practice and socio-demographic variables

Variable	COR (95% CI)	AOR (95% CI)
Gender		
Female	*Ref*	
Male	1.97 (1.36, 2.87)***	1.81 (1.01, 3.26)*
Age		
< 24	2.72 (1.49, 4.93)**	1.12 (0.43, 2.91)
25–34	1.35 (0.77, 2.38)	0.75 (0.36, 1.72)
35–44	1.30 (0.72, 2.33)	1.04 (0.48, 2.64)
45–54	1.34 (0.69, 2.59)	1.09 (0.49, 2.41)
> 55	*Ref*	
Marital status		
Single	2.30 (1.04, 5.13)*	1.34 (0.43, 4.21)
Married	1.00 (0.47, 2.15)	0.98 (0.37, 2.59)
Divorced	1.14 (0.45, 4.52)	1.03 (0.26, 4.06)
Widowed	*Ref*	
Educational Level		
Illiterate	*Ref*	
Primary	0.96 (0.34, 2.70)	1.96 (0.56, 6.88)
Junior	1.02 (0.38, 2.73)	1.62 (0.46, 5.72)
Secondary	1.54 (0.62, 3.79)	1.92 (0.55, 6.69)
College	2.60 (1.02, 6.62)*	2.80 (0.74, 10.63)
Occupation		
Governmental	*Ref*	
Private service	1.08 (0.56, 2.07)	1.06 (0.48, 2.32)
Self employed	1.07 (0.54, 2.10)	0.76 (0.33, 1.79)
Unemployed	1.17 (0.60, 2.28)	2.27 (0.95, 5.45)
House wife	0.65 (0.40, 1.04)	1.45 (0.67, 3.10)
Student	2.34 (1.09, 5.04)*	0.72 (0.24, 2.10)
Monthly income		
Non incomers	*Ref*	
Less than or equal to 1000	0.92 (0.54, 1.56)	*NA*
1001 to 2500	1.22 (0.78, 1.19)	*NA*
Greater than 2500	1.43 (0.75, 2.73)	*NA*
Knowledge		
Knowledgeable	*Ref*	
Non- knowledgeable	2.17 (1.30,3.61)**	2.13 (1.12, 4.05)*
Attitude		
Positive Attitude	*Ref*	
Negative attitude	6.56 (4.23,10.16)***	7.47 (4.54, 12.29)***

Note: *** = *p* < 0.001, ** = *p* < 0.01, * = *p* < 0.05, *AOR* = Adjusted Odd’s Ratio *CI* = Confidence Interval, *COR* = Crude Odd’s Ratio, *NA* = Not Applicable, *Ref* = Reference category

## Discussion

In this study, self-medication seeking behavior was found to be prevalent. This finding is comparable with findings of similar studies conducted in Sudan, Greece and Kenya [12–14]. It was however lower than reported in Indonesia, Ethiopia, Southern Spain, Slovenia and Lithuania [15–17] and higher than studies conducted in Pakistan, Saudi Arabia, Nigeria, Yemen, other Kenyan study and Sudanese [11, 19–25]. The difference in prevalence of self-medication with antibiotics might be due to differences in study design, community awareness, and definitions.

This study revealed that amoxicillin was the most frequently used antibiotic for self-medication. This might be explained by the fact that it is a well-known antibiotic to the community compared to other antibiotics and its ease of accessibility. The pharmacy retail outlets were found to be the main source for obtaining the antibiotics, targeted intervention is therefore recommended to halt the sale of antibiotics without prescription. During the conduction of this study, scheduling of medicines was not in place in Eritrea and thus, the public had easy access to medicines without prescription. To overcome the problem, strict regulation, and continuous public sensitization on rational use of antibiotics should be enforced.

It is however encouraging that majority of the study participants had a good understanding that antibiotics are indicated for bacterial infections (not viral infections). In contrary, studies conducted in Saudi Arabia and Indonesia reported a higher community belief that antibiotics work against viral infections [11, 15]. Most of the respondents in this study had the notion that self-medication with antibiotics is an inappropriate practice. That being said, the high prevalence of self-medication despite the positive attitude reflects further interventions are required to hammer the issue of antibiotic use without prescription by all available means.

Self-medication with antibiotics was significantly associated with male gender, inadequate knowledge and negative attitude. While the links between SMA and both inadequate knowledge and negative attitude are self explained, the difference in gender could be attributed to the work related injuries that males suffer more frequently than females as wound infections were the most reported complaints that required SMA in this study.

This result was found to be harmonious with some studies [18, 21, 27], and contradicts with other studies [22, 23] when it comes to gender. Some studies showed no significant association of socio-demographic factors with the practice of SMA [22, 23, 27] and other studies showed educational level [12, 22, 26], age, [12, 28, 29] and socioeconomic status [12, 26] to have statistically significantly associated with SMA.

## Strength and limitation

Accommodations such as collecting the data during the weekends and in the evening of the weekdays was done to include different sociodemographic dimensions evenly in number and hence, high response rate was obtained. Additionally, this study assessed the SMA practice for the past 12 months. This can be regarded as a strength but on the other hand, it has resulted in recall bias.

## Conclusion

Though majority of the respondents considered self-medication with antibiotics as inappropriate practice, their antibiotic seeking behavior without prescription was found to be prevalent. Respondents’ poor knowledge about antibiotics and antibiotic resistance as well as overestimating the power of antibiotics, and accessibility of antibiotics without prescriptions in retail outlets could be the possible drives for self-medication. Therefore, corrective measures such as enforcement of existing laws and scheduling of medicines to regulate their access to the public would protect consumers from misuse. Besides, healthcare professionals and media outlets should play their role in counselling consumers to refrain from use of antibiotics without prescription.

## Additional file

Additional file 1: Questionnaire for obtaining the prevalence of self-medication with antibiotics and associated factors in the community of Asmara, Eritrea. (DOCX 18 kb)

## Data Availability

The complete dataset used and/or analyzed during the current study are available from the corresponding author and can be accessed upon reasonable request.

## Abbreviations

ACHS: Asmara College of Health Sciences
ADRs: Adverse Drug Reactions
AMR: Antimicrobial Resistance
AOR: Adjusted Odds Ratio
CI: Confidence Interval
COR: Crude Odds Ratio
CSPro: Census and Survey Processing System
ERN: Eritrean Nakfa
IQR: Inter Quartile Range
MOH: Ministry of Health
RTIs: Respiratory Tract Infections
SM: Self-medication
SMA: Self-Medication with Antibiotic
SPSS: Statistical Package for Social Sciences
UTIs: Urinary Tract Infections
WHO: World Health Organization
