# Supplementary material for: Prevalence of self-medication with antibiotics and associated factors in the community of Asmara, Eritrea: a descriptive cross sectional survey
Source: BMC Public Health. 2019 Jun 10;19:726. doi: 10.1186/s12889-019-7020-x (PMC6558833; doi:10.1186/s12889-019-7020-x)
Supplement: Supplementary file 1 — Questionnaire for obtaining the prevalence of self-medication with antibiotics and associated factors in the community of Asmara, Eritrea. (DOCX 18 kb) [file 12889_2019_7020_MOESM1_ESM.docx]

**Informed consent form in English**

This study is aimed at determining the prevalence of self-medication with antibiotics among households in Asmara, Eritrea and the factors associated with it. Your **HONEST ANSWERS** are crucial for the successful execution of the study and the optimal planning of interventions thereafter to safeguard the health of our society by promoting rational use of drugs. You will not be required to write your name as it is confidential.

**Written Informed Consent Section** - *Please read carefully*

I have decided to participate in this research without any coercion.

**Signature:** ___________

Thank you for your willingness to participate in this study.

**Questionnaire**

**Section A: Socio demography**

**Instruction:** Circle or write the answer that corresponds with the best answer.

| No | Socio demographic questions | Answer | Score |
| --- | --- | --- | --- |
| 1. | Sex | 1. Male  **2**. Female |  |
| 2. | Age | . |  |
| 3. | Marital status | **1**. Married  **2**. Single  **3**. Divorced  **4**. Widowed |  |
| 4. | Level of education | **1.**Iliterate  **2.** Primary School **3**.JuniorSchool  **4**.High school  **5.**College |  |
| 5. | Occupation | **1**.Governmental  **2**. Private service  **3**.Self employed  **4.**Unemployed  **5.**Student  **6**.House wife |  |
| 6. | Monthly income | . . |  |

| **no** | **Statement** | **answer** | **score** |
| --- | --- | --- | --- |
| 1. | Have you ever taken antibiotics? | 1. Yes 2. No   If NO, please go Question no 13 |  |
| 2. | Have you ever treated yourself (self-medicated) with antibiotics? | A. Yes  B. No  If NO, please go to Question no 12 |  |
| 3. | How many times did you treat yourself with antibiotics in the past one year? | …………………………………. |  |
| 4. | What was (were) your reason(s) of self-medication with antibiotics? (check more than one if applicable | 1. Poor economic status 2. No access to physician care 3. Cost saving 4. Lack of trust in prescribing Doctor 5. Emergency use/ to get quick relief 6. The disease was not serious 7. Convenience of place 8. My own Previous successful experience 9. Others specify*___________________* |  |
| 5. | For which of the following complaint(s) did you use antibiotics? (check more than one if applicable) | A. Runny nose B. Nasal congestion  C. Cough. D. Sore throat  E. Fever. F. Aches and pains  G. Vomiting H. Diarrhea  I. Skin wounds. J. EAR/ EYE  K. Others specify.................................... |  |
| 6. | Your selection of antibiotics was based on  (check more than one if applicable) | 1. Pharmacist 2. Friends/ relatives 3. Leaflet 4. Internet/mobile applications 5. Previous doctor’s prescription 6. Others specify*___________________* |  |
|  | | | |

|  |
| --- |

| 8. | How did you know the dosage of antibiotics? (check more than one if applicable) | 1. By checking the package insert 2. By consulting a doctor 3. C. By consulting a pharmacist 4. D. By consulting family members/friends 5. E. From the newspapers, magazines, books, or TV programs   F. From the Internet  G. From my previous experience  H. By guessing the dosage by myself  I. Others..............(specify) |  |
| --- | --- | --- | --- |
| 9. | Did you ever change the dosage of antibiotics deliberately during the course of self-treatment? | A. Yes, always  B. Yes, sometimes  C. Never  If Never, please go to Question 10 |  |
| 10. | Why did you change the dosage of antibiotics during the course of self-treatment? (check more than one if applicable) | A. Improving conditions  B. Worsening conditions  C. To reduce adverse reactions  D. Drug insufficient for complete treatment  E. Others (specify) . |  |
| 11. | Please write down the names of antibiotics you have ever taken for SELF-MEDICATION: | A. B.  C. |  |
| 12. | When did you normally stop taking antibiotics? (check more than one if applicable) | A. After a few days regardless of the outcome  B. After symptoms disappeared  C. A few days after the recovery  D. After antibiotics ran out  E. At the completion of the course  F. After consulting a doctor/pharmacist  G. Others (specify) . . |  |
| 13. | What do you think about self-medication with antibiotics for self health care? | A. Good practice  B. Acceptable practice  C. Not acceptable practice |  |
| 14. | Do you know what are antibiotics? | A. Yes  B. No |  |
| 15. | What are antibiotics used for? (check more than one if applicable) | A. Virus infection  B. Bacterial infection  C. Others (specify) |  |
| 16. | Are antibiotics good for common cold? | 1. Yes. 2. No |  |
